# Clinical Importance of Amyloid Beta Implication in the Detection and Treatment of Alzheimer’s Disease

**DOI:** 10.3390/ijms26051935

**Published:** 2025-02-24

**Authors:** Justyna Pokrzyk, Agnieszka Kulczyńska-Przybik, Ewa Guzik-Makaruk, Izabela Winkel, Barbara Mroczko

**Affiliations:** 1 Department of Neurodegeneration Diagnostics, Medical University of Białystok, 15-269 Bialystok, Poland; justynapokrzyk@gmail.com (J.P.); mroczko@umb.edu.pl (B.M.); 2 Faculty of Law, University of Bialystok, 15-213 Białystok, Poland; ewa.guzik@tlen.pl; 3 Dementia Disorders Centre, Medical University of Wroclaw, 50-425 Ścinawa, Poland; i.winkel@me.com; 4 Department of Biochemical Diagnostics, Medical University of Białystok, 15-269 Bialystok, Poland

**Keywords:** amyloid beta, amyloid PET, amyloid blood, neurodegenerative disease, Alzheimer’s disease

## Abstract

The role of amyloid beta peptide (Aβ) in memory regulation has been a subject of substantial interest and debate in neuroscience, because of both physiological and clinical issues. Understanding the dual nature of Aβ in memory regulation is crucial for developing effective treatments for Alzheimer’s disease (AD). Moreover, accurate detection and quantification methods of Aβ isoforms have been tested for diagnostic purposes and therapeutic interventions. This review provides insight into the current knowledge about the methods of amyloid beta detection in vivo and in vitro by fluid tests and brain imaging methods (PET), which allow for preclinical recognition of the disease. Currently, the priority in the development of new therapies for Alzheimer’s disease has been given to potential changes in the progression of the disease. In light of increasing amounts of data, this review was focused on the diagnostic and therapeutic employment of amyloid beta in Alzheimer’s disease.

## 1. Introduction

### 1.1. Description of Amyloid Beta

Amyloid beta peptide (Aβ) is produced through proteolytic processing of amyloid precursor protein (APP) by enzymes, called β- and γ-secretases. APP is a membrane glycoprotein that plays a significant role in the field of biological activities, including intracellular transport or neuronal development [1]. Aβ peptides exist in different species as monomers, β-stranded fibrils, and multiple oligomers. Among the various Aβ forms, oligomers seem to be more toxic than fibrils [2]. A few APP cleavage products can cause some neuronal dysfunctions related to Alzheimer’s or Parkinson’s disease [3]. Deposits of Aβ peptides are located mainly in the hippocampus, neocortex, and cerebral vasculature [4]. Amyloid monomers aggregate into various types of conglomerates, but the most important are polymorphic fibrils. They are insoluble and may accumulate into amyloid plaques, the key neuropathological hallmarks in the brain of Alzheimer’s disease (AD) patients [5].

In addition to amyloid beta, several other proteins are associated with the pathological processes underlying Alzheimer’s disease. These proteins play various roles in the development and progression of the disease and are targeted for diagnostic and therapeutic purposes. Literature data indicate that besides amyloid beta also other crucial indicators of AD pathology are found including Tau protein, different forms of pTau proteins, apolipoprotein E (APOE), synaptic proteins, and neurotrophic factors (Table 1) [6,7].

The diagnosis and understanding of Alzheimer’s disease involve an assessment of multiple proteins and their interactions, reflecting the complex pathophysiology of the disorder. Those proteins offer potential targets for diagnostic, prognostic, and therapeutic interventions in AD [16,17].

### 1.2. The Role of Amyloid Beta in Memory Regulation

The physiological role of amyloid beta peptide in memory regulation has been a subject of considerable interest and debate in neuroscience research [18]. Recent findings have shed some light on the intricate relationship between Aβ and memory function, revealing a dual nature where low physiological doses enhance memory, whereas higher pathological doses impair it [19].

Studies on animal models have consistently demonstrated a hormetic effect of Aβ on memory. This phenomenon suggests that low doses of Aβ act as memory enhancers, whereas higher doses lead to cognitive impairment [20]. This biphasic response, known as hormesis, is a common feature in the regulation of various neurotransmitters, including acetylcholine [18,21]. A growing body of evidence implicates Aβ modulating mitochondrial function, influencing further memory processes. Aβ affects the mitochondrial function directly and indirectly, leading to mitochondrial dysfunction. Specifically, Aβ is transported into mitochondria where its accumulation disrupts the translocation of essential proteins involved in the mitochondrial function. Dysfunctional mitochondria, in turn, contribute to increased production of Aβ, establishing a bidirectional relationship between mitochondrial dysfunction and Aβ accumulation [22]. Another very important feature of Alzheimer’s disease pathology is disruption of the blood–brain barrier (BBB), although the findings regarding BBB dysfunction concerning Aβ clearance are inconsistent. While some studies suggest impaired Aβ efflux from the brain in Alzheimer’s disease, others fail to confirm a significant BBB disruption [23,24]. Nonetheless, central nervous system vascular disease, often associated with Alzheimer’s disease, remains a major factor in BBB dysfunction [25].

While individuals with Alzheimer’s disease exhibit an excess of amyloid beta protein, the relationship between amyloid beta load and cognitive dysfunction remains unclear. Studies suggest that amyloid plaques may not directly correlate with cognitive decline, as evidenced by plaque clearance without cognitive improvement, following immunization with amyloid beta. This underscores the importance of soluble Aβ species in memory regulation and cognitive function [26].

In conclusion, the physiological role of amyloid beta peptide extends beyond its association with Alzheimer’s disease pathology. Aβ exhibits a hormetic effect on memory, with low doses enhancing memory and higher doses impairing it. Furthermore, Aβ influences the mitochondrial function, BBB integrity, and memory processes. Understanding the dual nature of Aβ in memory regulation is crucial for developing effective treatments for Alzheimer’s disease, as interventions aimed at lowering Aβ levels must consider the delicate balance required for maintaining memory function. Additionally, the focus on soluble Aβ species underscores the complexity of Alzheimer’s disease pathology and the need for further research to elucidate the mechanisms underlying memory impairment [22,26].

## 2. Diagnostic Application of Amyloid Beta

The search for reliable biomarkers in Alzheimer’s disease is driven by the urgent necessity for early and accurate diagnosis, as well as for monitoring disease progression and response to treatment. Fluid biomarkers provide a non-invasive and accessible way to assess AD pathology, reflecting the underlying molecular changes in the brain. Amyloid beta isoforms have gained significant attention in neurology and cognitive science due to their association with Alzheimer’s disease pathology.

Amyloid beta diagnosis is important not only in Alzheimer’s disease but also in several other neurodegenerative disorders and conditions (Table 2). Accurate detection of amyloid beta deposition can facilitate early diagnosis, prognosis, and personalized treatment approaches in such conditions as Cerebral Amyloid Angiopathy (CAA) [27], Dementia with Lewy Bodies (DLB) [28], Frontotemporal Dementia (FTD) [29], Down Syndrome (DS) [30], and Traumatic Brain Injury (TBI) [31].

Advances in diagnostic techniques have facilitated the identification and characterization of various Aβ isoforms, offering valuable insights into the pathogenesis and progression of AD. Aβ peptides are derived from the proteolytic cleavage of amyloid precursor protein and exist in multiple isoforms, primarily categorized by their length. The most abundant and extensively studied variants are Aβ40 and Aβ42. Aβ42 has a greater tendency to aggregate, forming the amyloid plaques seen in AD brains, while Aβ40 is generally considered less amyloidogenic. The balance between these isoforms is critical in determining the onset and progression of AD pathology. Accurate detection and quantification of Aβ isoforms are essential for diagnostic purposes and therapeutic interventions [32]. Traditional diagnostic methods primarily rely on cerebrospinal fluid analysis, since Aβ peptides are readily detectable in this fluid and reflect the underlying brain pathology. However, distinguishing between different Aβ isoforms poses a considerable challenge due to their structural similarities and overlapping concentrations. By leveraging state-of-the-art mass spectrometry techniques coupled with immunoaffinity enrichment, the researchers demonstrated the feasibility of discriminating different Aβ isoforms with high precision and sensitivity [33]. This breakthrough methodology not only enhances our understanding of Aβ dynamics but also holds immense promise for improving AD diagnosis and monitoring. Recent advancements in assay technologies have facilitated the sensitive and specific quantification of CSF biomarkers, enabling their routine use in clinical settings. Moreover, standardized protocols and reference values as well as unique comments from results are established to ensure consistency and reproducibility across different laboratories and studies [33]. Recent research efforts have focused on exploring alternative biomarkers and imaging modalities for Aβ isoform detection, including positron emission tomography (PET) imaging with Aβ-specific ligands and blood-based biomarkers [34]. Such emerging diagnostic tools offer non-invasive and accessible means of assessing Aβ pathology, complementing conventional CSF analysis and facilitating early detection of AD-related changes [35].

### 2.1. Blood Amyloid Beta

Beyond CSF, blood-based biomarkers have garnered increasing interest due to their potential for minimally invasive and cost-effective diagnostic approaches. The potential for blood-based biomarkers for AD, including amyloid beta, has generated considerable interest due to the accessibility and minimally invasive nature of blood sampling. Plasma amyloid measurement is one of the key advancements in Alzheimer’s disease diagnostics. Blood-based biomarkers, such as amyloid beta, enable early and more accessible detection of AD without requiring expensive PET imaging or cerebrospinal fluid analysis [36,37]. The integration of blood biomarkers into AD diagnostics promises to revolutionize clinical care and research by allowing for more widely available diagnostic testing. Researchers’ attention has been focused on measuring not just Aβ 40 and 42, but also glial fibrillary (GFAP), neurofilament light (NfL), and phosphorylated tau (ptau181 and ptau217) in blood [37,38]. Recent reports have indicated that biomarkers detected from venous dried plasma spots (DPSvenous) could be helpful tools in the diagnosis and monitoring of neurodegenerative diseases [38]. It was established that GFAP, NfL, pTau181, and pTau217 in DPSvenous strongly correlated with standard measurements. Moreover, the clinical relevance of these biomarkers was confirmed by the fact that DPS biomarkers allowed for the detection of amyloid pathology and were associated with neurocognitive tests [38].

In the updated criteria, biomarkers are grouped into categories that reflect specific pathogenic processes. A core category includes biomarkers of AD neuropathologic change (ADNPC), encompassing amyloid biomarkers. This new approach distinguishes imaging biomarkers from fluid biomarkers like plasma, as plasma biomarkers reflect current rates of amyloid production and clearance, while imaging biomarkers (such as PET) indicate the cumulative effects of pathology in the brain. The 2018 classification scheme, amyloid/tau/neurodegeneration (AT(N)), has been expanded to include newly developed blood biomarkers. Additionally, new biomarker categories have been introduced, such as inflammatory markers, vascular markers, and alpha-synuclein markers, which often coexist with AD pathology in older patients. This updated framework enables the broad application of plasma amyloid measurement for purposes such as diagnosis, prognosis, disease staging, and monitoring treatment effects [39].

While significant progress has been made in blood-based biomarker research for AD, including amyloid beta, several challenges still remain. Standardization of assay protocols, validation in large and diverse cohorts, and replication across independent studies are crucial steps in developing and clinically implementing blood-based biomarkers for AD diagnosis and monitoring. It is worth noting that advancements in blood-based biomarkers for amyloid beta detection continue to evolve, and new research findings may provide further insights into their diagnostic utility and clinical significance [39].

Challenges for detection of amyloid beta, particularly in blood samples, were a low concentration of amyloid beta in blood and the presence of interfering substances, which complicate its measurement. However, the development of new technologies and analytical methods is a huge driving force contributing to significant progress in the field of Alzheimer’s disease researches.

Novel assay platforms which are emerging technologies, such as single-molecule array (SIMOA) assays, utilize ultra-sensitive detection methods to measure very low levels of proteins. SIMOA is an ultra-sensitive detection technique capable of identifying single molecules in femtoliter-sized wells, significantly improving sensitivity compared to traditional immunoassays. It allows the detection of amyloid beta at extremely low concentrations in plasma, making it a valuable tool in clinical applications. Those platforms hold promise for improving the early detection and monitoring of AD-related changes [36,40].

On the other hand, mass spectrometry techniques offer high sensitivity and specificity for detecting amyloid beta peptides in blood. Targeted mass spectrometry assays can quantify specific amyloid beta isoforms with high accuracy, although those methods may require sophisticated instrumentation and expertise [41].

Immunoprecipitation-Mass Spectrometry (IP-MS) combines immunoprecipitation to selectively isolate amyloid beta peptides with mass spectrometry for precise quantification. This method provides high specificity and sensitivity, making it suitable for clinical studies focused on analyzing different isoforms of amyloid beta. Despite its benefits, it requires specialized and expensive equipment, which limits its accessibility [42].

Surface Plasmon Resonance (SPR) enables label-free detection of amyloid beta by measuring changes in the refractive index near a sensor surface upon binding. This technique offers high sensitivity and specificity, making it a powerful tool for research on amyloid beta interactions. However, its requirement for expensive instrumentation and limited scalability pose challenges for widespread use [43,44].

Nanoparticle-based sensors utilize the conductive or optical properties of nanoparticles to detect amyloid beta. These sensors offer a fast, cost-effective, and highly sensitive detection method. Although still in the preclinical research phase, they hold promise for developing miniaturized and affordable diagnostic tools. However, their clinical implementation is still in development and not yet standardized [45].

Nanowire Field-Effect Transistor (FET) sensors detect amyloid beta through conductivity changes induced by molecular interactions at the nanowire surface. They offer ultra-high sensitivity and real-time detection capabilities, making them promising tools for future diagnostics. However, their variability in fabrication and susceptibility to environmental noise present challenges in achieving reproducibility and reliability [46].

Nanomechanical resonators detect amyloid beta by measuring frequency shifts resulting from molecular binding, allowing for mass-based detection. These sensors are highly sensitive and can detect extremely low concentrations of amyloid beta. However, their data analysis is limited, and precise environmental conditions are required to ensure accurate measurements [47].

Positron Emission Tomography (PET) imaging is a non-invasive method that uses radiolabeled ligands to visualize amyloid plaques in the brain [35]. This technique is highly specific for detecting fibrillar amyloid beta deposits and is widely used in clinical diagnostics. Nevertheless, its high cost and the requirement for specialized imaging facilities, along with radiation exposure, limit its accessibility for routine screening [48].

For amyloid pathologies, mutant forms of amyloid beta peptide must be well identified and detected, as these mutants have been linked to familial diseases. Specific mutations such as the Dutch mutation (E22Q), Arctic mutation (E22G), and Italian mutation (E22K) have been associated with early-onset Alzheimer’s disease [49]. The identification and detection of these mutants are essential for understanding their role in disease progression. Conventional techniques such as mass spectrometry can differentiate between wild-type and mutant amyloid beta peptides, while novel sensing schemes, including nanotechnology-based biosensors and high-resolution molecular imaging, offer enhanced sensitivity and specificity. The use of advanced analytical tools, such as computational modeling combined with AI-driven diagnostics, may further improve the ability to detect and characterize these mutations. As research progresses, refining these detection strategies will be critical for early diagnosis and targeted therapeutic interventions [50].

A variety of methods exist for detecting amyloid beta, each with distinct strengths and weaknesses (Table 3). While techniques such as SIMOA and IP-MS have already found clinical applications, emerging technologies like nanowire FET sensors and nanomechanical resonators offer new possibilities for highly sensitive detection. The selection of a method depends on factors such as cost, specificity, sensitivity, and clinical applicability. Further research and technological advancements are essential to improve these detection strategies and enhance the early diagnosis and monitoring of Alzheimer’s disease.

#### Factors Affecting the Amyloid Beta Assessment

It is important to mention some factors that could influence the aggregation of amyloid beta and other proteins. Firstly, temperature, because it can influence the aggregation kinetics of Aβ peptides. Higher temperatures generally accelerate the aggregation process, leading to faster formation of Aβ fibrils and aggregates. Conversely, lower temperatures may slow down aggregation kinetics. The temperature at which Aβ marking experiments are conducted can impact the rate and extent of Aβ aggregation and subsequent detection [51]. Secondly, the pH of the solution in which Aβ peptides are suspended can affect their aggregation behavior. Aβ aggregation is influenced by changes in pH, with optimal aggregation typically occurring at neutral to slightly acidic pH values. Extreme pH conditions (e.g., very low or very high pH) can destabilize Aβ peptides and alter their aggregation kinetics [52]. Thirdly, the ionic strength, determined by the concentration of salts in the solution, can modulate Aβ aggregation. Changes in ionic strength can affect the electrostatic interactions between Aβ peptides and influence their propensity to aggregate. Higher ionic strength may promote Aβ aggregation by screening repulsive forces between peptides, while lower ionic strength may destabilize Aβ aggregates [53]. Another factor is the presence of aggregation inhibitors or enhancers, because various molecules, including small molecules, peptides, and antibodies, can modulate Aβ aggregation. Aggregation inhibitors, such as certain small molecules or antibodies targeting Aβ, can either prevent or slow down Aβ aggregation. Conversely, aggregation enhancers or nucleating agents may accelerate Aβ aggregation and fibril formation [54]. Then, metal ions, such as copper, zinc, and iron, can interact with Aβ peptides and influence their aggregation and toxicity. Metal ions can accelerate Aβ aggregation by promoting peptide oligomerization and fibril formation. Additionally, metal-Aβ interactions may generate reactive oxygen species (ROS) and oxidative stress, contributing to neuronal damage in Alzheimer’s disease. Another important issue is protein concentration, because the concentration of Aβ peptides in solution can impact their aggregation kinetics and the morphology of resulting aggregates. Higher Aβ concentrations typically lead to faster aggregation kinetics and the formation of larger aggregates. However, very high concentrations of Aβ may also result in nonspecific aggregation and the formation of amorphous aggregates [55]. The last factor is sample handling and storage. Handling and storage conditions of Aβ samples can influence their state of aggregation and stability. Factors such as the sample storage temperature, duration, and exposure to light or air can affect the integrity of Aβ peptides and their propensity to aggregate. Proper sample handling and storage protocols are essential to maintain the reproducibility and reliability of Aβ marking experiments [56].

### 2.2. Clinical Utility of Amyloid PET

In the diagnostics process, PET plays a very important role. A thorough examination is possible with Pittsburgh Compound B (PIB) which is a valuable tool for visualizing and quantifying cerebral Aβ pathology in vivo. PIB is currently the most extensively studied and utilized tracer for PET imaging of Aβ pathology in the brain. Studies have consistently shown a significantly higher binding of PIB in the brains of individuals with symptomatic AD compared to cognitively normal (CN) older adults. This binding pattern follows a specific distribution in the brain, which is helpful in the diagnosis and understanding of AD pathology. PIB exhibits selectivity for fibrillar Aβ and shows a high affinity to a single binding site in brain tissue samples from AD patients. It is worth noting that PIB specifically binds to Aβ-40 and Aβ-42 synthetic fibrils and insoluble Aβ plaques containing those fibrils found in AD brains. However, it does not appreciably bind to soluble Aβ or oligomeric forms, requiring an extended Aβ pleated sheet structure for high-affinity binding [57]. Moreover, it demonstrates minimal binding to the cerebellum, making it suitable for reference region-based quantification. Quantitative assessment of PIB uptake can be performed by using various methods, including distribution volume ratios (DVR) or binding potentials (BP) obtained through the Logan graphic method with the cerebellum as the reference region. Alternatively, standardized uptake value ratios (SUVRs) can be calculated manually or with automatic algorithms, representing the ratio of PIB accumulation in specific cortical regions to its retention in the entire brain. Recent advancements include the development of an image-derived input function approach for quantitative assessment of PIB retention without the need for arterial sampling [58]. Additionally, other techniques for quantification are under active development and are aimed at enhancing the accuracy and efficiency of PET imaging for Aβ pathology assessment in AD and related disorders. Those advancements hold promise for improving early diagnosis, monitoring disease progression, and evaluating therapeutic interventions in AD. Regional analysis of PET images is typically conducted by using manually defined regions of interest based on co-registered MR images. Semi-automatic and automatic methods, as well as voxel-wise analyses such as Statistical Parametric Mapping (SPM), are also employed. However, different centers may use various global means of representative cortical regions, leading to difficulties in cross-center comparisons. For example, in some institutions, the mean cortical binding potential (MCBP) is calculated from the prefrontal cortex, gyrus rectus, lateral temporal, and precuneus regions. Others may use different combinations of regions such as frontal, anterior cingulate, precuneus, lateral temporal, parietal cortex, and striatum [58]. There are also some variations in the calculation of average cortical SUVR which may involve area-weighted means of specific cortical regions. Recent developments involve the synthesis and experimental evaluation of 18F-labeled PIB analogs aimed to improve PET imaging capabilities for Aβ pathology. Those advancements contribute to our understanding and detection of Aβ-related changes in the brain, facilitating early diagnosis and therapeutic intervention in Alzheimer’s disease and related conditions [48].

#### Amyloid PET in the Early Detection of Disease

The concept of preclinical Alzheimer’s disease suggests that AD-related lesions accumulate in the brain for years before the onset of cognitive deficits or dementia symptoms. It is postulated that individuals with preclinical AD may eventually progress to clinical dementia due to AD, although the timeframe for this progression can vary according to individual reserve capacities [34]. Postmortem analyses have shown that even cognitively normal individuals can harbor significant densities of senile plaques and Aβ immunohistochemistry in their neocortex, with some individuals exhibiting extensive neocortical plaque distribution suggestive of a preclinical stage of AD. This suggests that the AD disease process may begin well before the clinical manifestation of dementia. Current recommendations propose a continuum or trajectory model for understanding AD, where both underlying pathological processes and clinical symptoms evolve over time, possibly in parallel but temporally offset trajectories [58]. This concept led to the definition of three staging categories for preclinical AD research, each representing different levels of biomarker evidence and clinical symptoms. Imaging and molecular biomarkers, such as PIB PET imaging, have become crucial in identifying in vivo correlates of neuropathological AD and markers of preclinical AD. Elevated PIB uptake, indicative of Aβ accumulation, has been consistently observed in CN older individuals, suggesting it as an important in vivo pathological hallmark of preclinical AD. Recent studies have proved a relatively high frequency of elevated PIB binding in CN adults, particularly in older individuals, comparable to the age-related frequency of neuropathological AD observed postmortem. The spatial distribution of PIB uptake in CN older individuals mirrors that observed in AD, with elevated uptake in regions such as the posterior cingulate, precuneus, gyrus rectus, orbitofrontal, prefrontal, and lateral temporal cortex. Notably, CN individuals typically exhibit similar or lower levels of PIB uptake compared to those with mild cognitive impairment or DAT. These findings underscore the importance of using biomarkers, such as PIB PET imaging, to detect early pathological changes in the brain associated with AD, facilitating early diagnosis and potentially informing interventions aimed at delaying or preventing the onset of clinical symptoms [57].

Autosomal dominant Alzheimer’s disease has garnered significant attention due to its ability to allow the evaluation of amyloid beta accumulation in young and middle-aged adults with known mutations many years or even decades before the onset of clinical symptoms. Studies that make use of PIB PET imaging in individuals with these mutations have revealed evidence of regional Aβ deposition, particularly in such areas as the precuneus, posterior cingulate, prefrontal cortex, and striatum. Remarkably, those findings have been observed in carriers who were up to 10 years younger than the typical age of onset for their family’s AD. Longitudinal studies, employing PIB PET as an indicator of Aβ accumulation, have provided valuable insights into the progression of AD pathology. It has been observed that individuals with mild cognitive impairment and Alzheimer’s dementia show either no or minimal increases in tracer uptake during 1–3 years of follow-up. This suggests that brain Aβ accumulates more rapidly during the early phases of AD and progresses slowly during the more advanced stages of the disease. Moreover, recent longitudinal PIB studies have demonstrated an increase in Aβ accumulation over time in cognitively normal individuals with elevated baseline levels of Aβ. These findings highlight the importance of monitoring Aβ accumulation in preclinical and prodromal stages of AD, as it could provide crucial insights into disease progression and become helpful in the development of early interventions [48].

In addition to age, the genetic background plays a significant role in Alzheimer’s disease risk, with the APOE ε4 allele being the major genetic susceptibility factor for late-onset AD. The risk associated with APOE ε4 is gene dose-dependent, meaning individuals carrying more copies of the ε4 allele have a higher risk of developing Alzheimer’s at an earlier age. Conversely, other isoforms of APOE, such as APOE ε3, are considered neutral, while APOE ε2 may even confer protective effects against AD. APOE ε4 carriers have been shown to have increased cerebral amyloid deposition compared to non-carriers, and this risk appears to be directly related to its effect on AD pathology [9].

Preclinical AD, characterized by the presence of AD pathology in cognitively normal individuals, is increasingly recognized as a precursor to symptomatic AD. Individuals with preclinical AD may exhibit reduced levels of CSF Aβ42, which are associated with whole-brain atrophy and hypometabolism in the medial temporal lobe. Moreover, those with elevated PIB binding levels, indicative of amyloid deposition, may demonstrate multiregional brain atrophy, cortical thinning, and functional MRI connectivity deficits similar to AD, along with episodic memory deficits and longitudinal cognitive decline. Notably, individuals with elevated mean cortical binding potential are at significantly greater risk of developing symptomatic AD compared to those with lower levels of amyloid deposition [34]. Moving forward, careful longitudinal studies with multiple repeated imaging and diagnostic assessments are needed to better understand the onset and progression of AD pathology. Longitudinal data are crucial for characterizing the conversion from healthy aging to preclinical AD and for developing reliable tools for early detection and intervention. Moreover, collaborative efforts between institutions, such as the Alzheimer’s Disease Neuroimaging Initiative (ADNI) and the Dominantly Inherited Alzheimer’s Network (DIAN), are essential for advancing research and clinical trials in AD, particularly in populations with autosomal dominant AD. Such initiatives enable the pooling of resources and data from multiple centers, facilitating adequately powered longitudinal studies and clinical trials in these unique and informative populations [59]. 

## 3. Therapeutic Strategies

Neurodegenerative diseases have limited therapeutic options. Available treatment allows only for suppressing the symptoms. We have reviewed novel therapeutic approaches in five categories: anti-amyloid therapy, anti-tau therapy, anti-neuroinflammatory therapy, neuroprotective agents including N-methyl-D-aspartate (NMDA) receptor modulators, and brain stimulation [60].

### 3.1. Therapeutic Strategies Targeting Amyloid Beta

**Figure 1:**
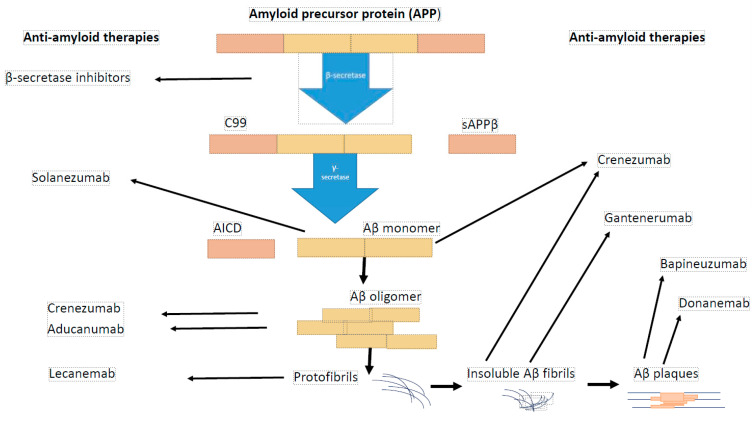
Tested immunotherapy against amyloid beta.

It was revealed that amyloid beta is present in various species, including monomers, oligomers, protofibrils, and insoluble fibrils in plaques. Oligomers and protofibrils are toxic, and removal of these aggregates might represent an effective treatment for AD. Anti-amyloid therapy consists of three strategies: Aβ immunotherapy (such as aducanumab and other types of antibodies—Figure 1), secretase inhibitors, and Aβ aggregation inhibitors (Table 4).

Immunotherapy against amyloid beta is a promising type of treatment for Alzheimer’s disease. The U.S. Food and Drug Administration (FDA) has approved three anti-amyloid antibodies for Alzheimer’s disease including aducanumab (Aduhelm—2021), lecanemab (Leqembi—2023), donanemab (Kisunla—2024); however, none of these drugs have been registered as routine treatments for AD patients in Europe [61]. Aducanumab is a monoclonal antibody targeted at aggregated soluble and insoluble forms of amyloid beta. As intended, aducanumab slows down the progression of the disease. It does not reverse any already existing damages, so it is targeted at the early stage of Alzheimer’s disease and prodromal patients (amyloid-positive by PET, but asymptomatic). According to three randomized, controlled trials aducanumab reduced brain amyloid beta plaques in a time- and dose-dependent manner (3 mg/kg, 6 mg/kg). Moreover, the cognitive impairment was slower in the high-dose treated group (10 mg/kg). A cognitive improvement was noticed in patients with mild-to-moderate AD. Levels of plaque were monitored using positron emission tomography. Because of some side effects connected with amyloid-related imaging abnormalities, such as temporary edema in some areas of the brain, testing of Aducanumab’s efficacy and safety is necessary to corroborate that any benefit from this drug would outweigh the risk. Nevertheless, although there were usually no symptoms observed after aducanumab therapy, some patients suffered from vision changes, nausea, confusion or headaches. On the other hand, there was a risk of hypersensitivity reactions, such as angioedema or urticaria [62,63]. Lecanemab is a humanized IgG1 monoclonal antibody directed against soluble aggregated Aβ species (protofibrils) which has shown intense brain fibrillar amyloid reduction and amelioration of clinical decline in early AD [64]. Data from a randomized and double-blind study have revealed that participants who were treated with lecanemab at a dose of 10 mg/kg twice a week for 12 and 18 months demonstrated dose-dependent decrease of brain amyloid measured PET and corresponding improvements in plasma Aβ42/40 ratio, and reductions in plasma p-tau181 changes and slowing of cognitive decline. Furthermore, when the administration of lecanemab was discontinued, the rates of disease progression were similar in lecanemab and placebo patients; however, better treatment results were noticed in the group previously treated with lecanemab. Additionally, superior diagnostic performance in the assessment of progression was noticed for plasma Aβ42/40 ratio, and p-tau181 (earlier indicators) compared to amyloid PET [64].

Data reported in the literature indicate that other monoclonal antibodies directed against amyloid beta, including solanezumab, crenezumab, gantenerumab, and bapineuzumab, failed to slow cognitive decline in AD patients in contrast to the three mentioned above [65,66]. Despite the encouraging outcomes from experiments on animal AD models and phase I and II clinical trials, solanezumab, an antibody recognizing and binding to the mid domain of Aβ peptide resulting in clearance of soluble Aβ, failed as an effective treatment for AD patients [67,68]. Investigations in a mouse AD model have demonstrated that solanezumab increases concentrations of Aβ in plasma and inhibits the deposition of Aβ plaque in mouse brains [69]. Moreover, phase I and II trial reports have revealed a good safety profile and elevated total Aβ concentrations in cerebrospinal fluid and plasma of AD patients [70]. However, a phase III clinical trial conducted in almost 1200 AD patients treated with solanezumab did not confirm that this passive immunotherapy offers a treatment benefit to AD patients. Solanezumab administrated intravenously (up to 1600 mg) every 4 weeks for 240 weeks did not attenuate the progressive cognitive decline in AD patients who received it (based on Preclinical Alzheimer Cognitive Composite (PACC) score), and it did not decrease amyloid levels in AD patients’ brains (based on 18F-forbetapir PET), as compared to the placebo group [68]. In agreement with those results are findings from phase III investigations on crenezumab, a humanized monoclonal immunoglobulin G4 antibody targeting amyloid beta oligomers, which revealed that it was well-tolerated but did not alleviate clinical decline in patients with early AD [71]. The assessment of the efficacy and safety of bapineuzumab in the treatment of mild-to-moderate Alzheimer’s disease were performed in two separate clinical trials in phase III. It was noticed that bapineuzumab did not improve clinical outcomes in AD patients, although treatment differences in biomarkers were observed in APOE ε4 carriers [72].

#### 3.1.1. β- and γ-Secretase Inhibitors

Secretase inhibitors restrain catalytic activities of β-secretase (BACE inhibitors) and γ-secretase, which reduces Aβ production. A decrease of amyloid beta levels in cerebrospinal fluid is observed while using some BACE inhibitors [73]. However, several BACE inhibitors are ineffective or even can aggravate cognitive function in patients with mild cognitive impairment, and mild to moderate AD. When it comes to γ-secretase inhibitors, cognitive deterioration was noted in patients with mild to moderate AD. Therefore, the role of secretase inhibitors remains under debate [73].

#### 3.1.2. Amyloid Beta Aggregation Inhibitors

Another option is amyloid beta aggregation inhibitors; however, they have a lot of disadvantages. Firstly, some compounds have little permeability across the blood–brain barrier. Secondly, those compounds are too tiny to disrupt Aβ aggregation. Thirdly, the protein–protein binding regions are relatively featureless to small molecules without specific receptors. A noteworthy strategy is to target the chaperones in the brain, such as metals. Interactions between Aβ peptides and metals are deranged, which causes a barrier against Aβ oligomerization. Metal protein-attenuating compounds (MPACs) chelate zinc and cooper ions and inhibit Aβ aggregation [74,75]. The results of some studies concerning these compounds’ treatment effectiveness are not unambiguous. A few studies showed that one of those substances, called clioquinol, which is a hydroxyquinoline ionophore, rescued cognitive decline in the more severely affected Alzheimer’s patients. In general, the therapy was safe and well-tolerated, but double-blind randomized controlled trials of second-generation clioquinol demonstrated no overall significant effect either on cognition or functions in treating mild to moderate AD. The latest experiments have been used to identify new potential compounds. Some of them increase Aβ aggregation-inhibitory activities, including uncarinic acid C and Tanshinone. It gives hope for potential therapeutic effects [74].

**Table 4:** Therapies targeting amyloid beta pathology.

Agent	Administration	Mechanism of Action	Indication	Clinical Status	Ref.
Aducanumab	Intravenously (IV)	Monoclonal antibody against Aβ oligomers. Removes Aβ	MCI, mild-to-moderate AD	In the US, approved by FDA	[76]
Solanezumab	Intravenously (IV)	Monoclonal antibody against Aβ monomers	MCI	Phase III completed, withdrawn	[68]
Donanemab	Intravenously (IV)	Monoclonal antibody against Aβ plaques	MCI	In the US, approved by FDA	[77,78]
Lecanemab	Intravenously (IV)	Monoclonal antibody against Aβ protofibrils	MCI	In the US, approved by FDA	[79]
BACE inhibitors (Lanabecestat)	Oral	Block the activity of BACE1, preventing the initial cleavage of APP	MCI	Discontinued from clinical trials (phase III and phase II) due to lack of efficacy in later stages of the disease	[80]
BACE inhibitors (Verubecestat)	Oral	Block the activity of BACE1, preventing the initial cleavage of APP	Mild-to-moderate AD patients	Withdrawn from clinical trials (phase III) due to worse daily functioning among treated patients vs. placebo	[80,81]
γ-secretase inhibitors (Semagacestat)	Oral	Inhibition of γ-secretase prevents the formation of amyloid beta peptides	AD	Discontinued in phase III clinical trial (semagacestat)—did not improve cognitive status and caused more adverse events (skin cancers and infections)	[82]
γ-secretase inhibitors (Avagacestat)	Oral	Inhibition of γ-secretase prevents the formation of amyloid beta peptides	AD	Discontinued in phase II clinical trial (Avagacestat-BMS-708163): did not demonstrate efficacy and was associated with adverse dose-limiting effects	[83]
Amyloid beta aggregation inhibitors (Clioquinol)	Oral or intravenous	These inhibitors bind to amyloid beta monomers, oligomers, or fibrils, blocking their aggregation or promoting disaggregation	Early stages of AD	Discontinued in phase II and phase III due to no significant effect of clioquinol versus placebo on the rate of cognitive decline in a cohort of patients with AD	[74]

### 3.2. Therapeutic Strategies Targeting Tau

Anti-tau therapy constitutes another very complex group of treatments directed at diminishing the pathological changes caused by tau protein in different neurodegenerative diseases. The group of anti-tau medications includes phosphatase modifiers, kinase inhibitors, tau aggregation inhibitors, microtubule stabilizers, and tau immunotherapy (Table 5). Sodium selenate is a phosphatase modifier that decreases phosphorylation by activating phosphatases, such as protein phosphatase 2A (PP2A). It is also an important molecule in neurophysiology. The studies have demonstrated that a deficiency of this compound is connected with oxidative damage and cognitive impairment. Unfortunately, it was revealed that sodium selenate supplementation had minor effects in mild to moderate AD [84].

#### 3.2.1. Kinase Inhibitors

Kinase inhibitors limit the hyper-phosphorylation of tau and decrease post-translational processes. The phosphorylation is related to protein kinases such as cyclin-dependent-like kinase 5 and glycogen synthase kinase-3β. Selective inhibitors of cyclin–dependent-like kinase 5 are not very successful in AD treatment. They are used in oncological therapy. Lithium, a mood stabilizer identified as a glycogen synthase kinase-3β inhibitor, was also assessed in AD treatment. Trials have shown that microdoses of lithium prevent cognitive decline (Table 5). The patients received a 15-month treatment, with a daily dose of 300 mg. Analyses corroborated that lithium inhibited the progression of cognitive decline in AD patients, with a moderate effect. However, lithium’s effectiveness in treatment should be further investigated [60].

#### 3.2.2. Tau Aggregation Inhibitors

Methylene blue (MB) was the earliest inhibitor of tau aggregation. In laboratory studies, MB has demonstrated the ability to block interactions among tau molecules and disrupt their polymerization. A phase II double-blind randomized controlled trial (RCT), involving a 50-week administration of 138 mg of MB, showed promising cognitive benefits in individuals with mild to moderate Alzheimer’s disease. Further research revealed that while MB inhibited tau fibril formation, it paradoxically accelerated the formation of neurotoxic tau oligomers, leaving its role in AD therapy uncertain [90].

Curcumin, a natural coloring agent and food additive, has also shown potential as a tau aggregation inhibitor. In laboratory settings, curcumin has been found to inhibit tau aggregation by reducing β-sheet formation in tau and disintegrating tau oligomers. Despite those promising findings, several phase II double-blind RCTs investigating curcumin’s efficacy in AD patients have failed to demonstrate any clinical or biomarker improvements, following a six-month treatment regimen. One of the major challenges attributed to the failure of curcumin studies is its low bioavailability. However, in cognitively healthy elderly individuals, curcumin has shown short-term improvements in working memory, following an acute administration (<4 weeks). Interestingly, long-term administration of curcumin did not yield significant deterioration in cognitive decline. Moreover, a recent systematic meta-analysis has suggested that curcumin treatment may even worsen cognitive performance in AD patients. Those findings underline the complexity of curcumin’s therapeutic potential in AD and highlight the need for further research to elucidate its mechanisms of action and optimize its clinical utility [91].

#### 3.2.3. Tau Immunotherapy

Active tau vaccines have been developed to elicit antibodies against tau proteins. Two such vaccines, AADvac1 and ACI-35, have progressed to clinical trials. AADvac1 generates antibodies targeting the microtubule-binding region of tau, which has been shown to decrease tau aggregation and promote tau clearance. In a phase I double-blind randomized controlled trial involving individuals with mild to moderate Alzheimer’s disease, nearly all patients who received AADvac1 injections exhibited an IgG immune response within 12 weeks. Importantly, no cases of meningoencephalitis or vasogenic edema were reported during a 72-week follow-up assessment. A phase II double-blind RCT of AADvac1 efficacy in mild AD patients has also been conducted [87,88,92].

ACI-35 is a liposome-based vaccine targeting phosphorylated tau. Animal studies have demonstrated that ACI-35 induces a rapid immune response and reduces phosphorylated tau levels in tau transgenic mice within 12 weeks. A phase II double-blind clinical trial assessed the tolerability and safety of the ACI-35 vaccine in patients with mild to moderate AD; however, no relevant differences between patients with mild or moderate Alzheimer’s disease and placebo were detected [88].

Passive immunotherapy for tau pathology has also made significant strides with several agents advancing to clinical trials for AD. They are humanized lgG4 or lgG1 monoclonal antibodies targeting tau. Gosuranemab (BIIB092) demonstrated safety and tolerability in healthy participants. Results of a phase II double-blind RCT reported that BIIB092 was well tolerated in participants with mild AD dementia. These advancements in active and passive immunotherapies represent promising avenues for the treatment of AD [89].

### 3.3. Other Methods of AD Treatment in Trials

#### 3.3.1. Anti-Neuroinflammatory Therapy

Neuroinflammation plays a significant role in the progression of Alzheimer’s disease and is closely associated with disease severity. Strategies aimed at mitigating neuroinflammation encompass a variety of approaches, including modulation of microglia and astrocytes, management of insulin resistance and microbiome therapy (Table 6) [60].

#### 3.3.2. Microglia Modulators

Activation of microglia constitutes a pivotal feature in neuroinflammation, intimately involved in the pathogenesis of Alzheimer’s disease. Microglia engage with amyloid beta and tau protein, key players in AD progression. This activation is intertwined with various signaling pathways, including apolipoprotein E, triggering receptor expressed on myeloid cells 2 (TREM2), Toll-like receptor (TLR), and colony-stimulating factor-1 receptor (CSF1R). Mutations in APOE and TREM2 are identified as potent risk factors for AD, and their pathway shares mechanisms in regulating Aβ pathology. APOE enhances TREM2-mediated phagocytosis of apoptotic neurons, leading to improved memory performance in AD mouse models. Conversely, deficiency in TREM2 exacerbates Aβ pathology. Despite their significance, no agents targeting APOE or TREM2 have advanced to clinical trials for AD treatment.

Several toll-like receptor pathways respond to Aβ accumulation, particularly TLR4 and TLR2. The TLR4 pathway interacts with NLRP3 inflammasomes, sustaining neuroinflammation, and contributes to memory impairment in AD models. Inhibitors of TLR4 have shown promise in ameliorating cognitive deficits in AD animal models. TLR2 mediates Aβ phagocytosis by microglia, and dysregulation of this pathway exacerbates memory impairment in AD mice. However, none of those agents has reached clinical trials for AD therapy.

The colony-stimulating factor-1 receptor pathway drives microglial proliferation in AD animal models. Selective CSF1R inhibitors have demonstrated efficacy in blocking microglial proliferation and improving memory deficits in various AD mouse models. Those inhibitors hold potential for therapeutic intervention in AD, offering hope for mitigating neuroinflammation and associated cognitive decline [60,104].

#### 3.3.3. Astrocyte Modulators

The astrocyte response plays a crucial role in impairing the clearance of Aβ at the blood–brain barrier in Alzheimer’s disease. Several signaling pathways underlie astrocyte reactivity in AD, including activation of transcription 3 (JAK/STAT3), calcineurin/nuclear factor of activated T cells (calcineurin/NFAT), nuclear factor-kB/nod-like receptor family pyrin domain containing 3 (NFκB/NLRP3), mitogen-activated protein kinase (MAPK), and the P2Y1 purinoreceptor (P2Y1R) pathways.

Activation of the JAK/STAT3 pathway has been observed in reactive astrocyte transgenic mouse models of AD. A selective STAT3 inhibitor has shown promising results in rescuing learning and memory deficits in AD mouse models. Similarly, inhibition of the calcineurin/NFAT pathway by FK506 (Tacrolimus) has demonstrated cognitive improvement in preclinical studies. Clinical investigations of FK506 are underway to evaluate its efficacy in mild cognitive impairment and AD.

The NFκB/NLRP3 pathway, activated by Aβ, contributes to the production of proinflammatory cytokines. Although targeting this pathway holds therapeutic potential, no agent has progressed yet towards clinical trials for AD treatment.

Furthermore, modulation of the P2Y1 purinoreceptor pathway has emerged as a promising strategy. Inhibition of P2Y1R has normalized astrocyte activity and ameliorated cognitive deficits in AD mouse models. Those findings underscore the potential of astrocyte modulators as therapeutic interventions in AD, warranting further clinical investigations to validate their efficacy and safety in human subjects [60].

#### 3.3.4. Insulin Resistance Management

Alzheimer’s disease is characterized by disturbances in cerebral glucose utilization, leading to progressive cognitive impairment. These deficits involve insulin deficiency, insulin-like growth factor 1 (IGF-1) deficiency, and insulin resistance, which exacerbate oxidative stress, inflammation, tau phosphorylation, and toxic amyloid beta levels [105,106].

Insulin therapy, administered intranasally, has shown promise in treating AD. Clinical trials have reported improvements in memory impairment among patients with mild cognitive impairment and AD following intranasal insulin administration. However, recent trials have yielded mixed results, with some showing no significant difference in improvement of cognition function compared to placebo. Despite this, intranasal insulin therapy appears relatively safe, with no serious adverse events reported.

Incretins, such as glucagon-like peptide-1 (GLP-1) and glucose-dependent insulinotropic polypeptide (GIP), stimulate insulin secretion and have shown therapeutic potential in animal models of AD. Liraglutide, an incretin receptor agonist, has demonstrated cognitive benefits in AD patients in clinical trials. Similarly, metformin, a first-line therapy for diabetes mellitus, has exhibited neuroprotective effects and memory preservation in MCI patients, irrespective of diabetes status.

Peroxisome proliferator activator receptors (PPARs) mediate anti-inflammatory and metabolic pathways implicated in AD pathogenesis. Pioglitazone, a PPAR-γ agonist, has demonstrated cognitive benefits in diabetic patients with mild AD but showed no efficacy in MCI patients in phase III trials [107].

#### 3.3.5. Microbiome Therapy

An intricate interplay between the gut microbiota and brain function emphasize the significance of gut-brain communication. The gut microbiota, by synthesizing various neurotransmitters and neuromodulators, play a pivotal role in shaping brain function. However, dysbiosis of the gut microbiota disrupts this delicate balance, leading to overproduction of lipopolysaccharides (LPS) in the gut and increased permeability of the blood–brain barrier [108].

Sodium oligomannate (GV-971), derived from marine sources, emerges as a promising intervention for gut microbiota dysbiosis. It works by suppressing dysbiosis, regulating neuroinflammation, and destabilizing amyloid beta aggregates. Phase III double-blind randomized controlled trials of sodium oligomannate have demonstrated cognitive benefits in patients with mild to moderate Alzheimer’s disease. Participants receiving a dose of 900 mg of sodium oligomannate for 36 weeks showed significant improvements in ADAS-cog performance compared to the placebo group. As a result, sodium oligomannate received approval in November 2019 in China for the treatment of mild to moderate AD [109].

#### 3.3.6. Role of Artificial Intelligence in Diagnosis and Treatment of AD

Increasing amounts of information about the patients (multiome datasets) including genetics, genomics, epigenomics, transcriptomics, proteomics, metabolomics, lipidomics, and radiomics, on the one hand, could be very helpful; however, on the other, it could increase the difficulty of personalized clinical diagnosis and treatment of AD [110]. In light of the above-mentioned facts, it seems crucial to apply software using artificial intelligence (AI) tools, such as machine learning, to the interpretation of such an enormous amount of data; this would permit development of algorithmic models from research data to prepare practical solutions that are accessible and easy to use [111]. Currently, artificial intelligence (AI) is a new tool in drug discovery and development for AD and other neurodegenerative diseases [112]. AI allows for de novo drug design by aiding in the design of molecules with desired properties, such as high binding affinity with protein targets and low predicted toxicity. Additionally, it enables calculation of a drug’s mechanism of action, virtual screening, and prediction of drug-target interactions [110,112]. AI also holds promise for development of early and precise Alzheimer’s disease diagnosis. It is suggested that eye movement analysis by deep learning and machine learning may identify cognitive processes and neuropathology in Alzheimer’s disease, therefore it could serve as an early and non-invasive diagnostic tool for patients with suspected Alzheimer’s disease [113]. It is worth stressing that AI may improve early and accurate diagnosis due to combining various patient data, including neuroimaging results, such as positron emission tomography and magnetic resonance imaging, blood-based and CSF biomarker results, and genetic data [114]. Furthermore, it is suggested that in the future it might be used for prevention of neurodegenerative diseases, and for customizing diets for people to decrease dementia risk factors, thus maintaining brain function and overall health [111].

## 4. Selected Legal Aspects of the Diagnostic and Therapeutic Implications of Amyloid Beta in Neurodegenerative Diseases

Human memory and mental well-being are a condition for being subject to law. Diagnostic and therapeutic implications of amyloid beta in neurodegenerative diseases should also be considered at the legal level. This applies to all areas of law, especially civil law and criminal law.

Alzheimer’s disease is the most common cause of dementia in older people and occurs in almost 70% of all dementia cases. Worldwide, 55 million people are living with Alzheimer’s and other dementias [43]. Based on some legal regulations, it can be assumed that people in the 1st phase of Alzheimer’s disease have full legal capacity. During that phase, there are no presumptions that would warrant declaring the person to lack capacity. The afflicted persons are aware of their behavior and are capable of controlling it. During the 2nd phase of AD, which involves mild cognitive impairment, the presumptions for lacking capacity do not necessarily determine full legal incapacity. It depends on the strength of the symptoms and their influence on the ability of the afflicted persons to control their actions. Declaration of legal incapacity and establishment of a custodian during that phase is in some cases justified. It is justified by the patients’ awareness, limitation of their actions, and by their limited control over their own behavior. The last phase of AD is a severe mental decline. During that phase, the presumptions for a lack of legal capacity are justified, as is the establishment of a custodian. The afflicted persons are not aware of their actions and have no control over their behavior [115]. Neurodegenerative diseases, including Alzheimer’s disease, are also relevant in criminal law. These disorders can define the perpetrator as insane. Patients in the third stage of Alzheimer’s disease are usually insane, hence they can be both the perpetrator and the victim of a criminal act.

Given these legal aspects, it seems that finding biochemical or radiological markers that will be useful in the early diagnosis of Alzheimer’s disease is important not only from a medical, but also from a legal point of view.

## 5. Conclusions

The role of amyloid beta is crucial in some physiological processes in the brain, including memory, as well as pathological mechanisms underlying neurodegenerative diseases. New precise methods of amyloid beta detection improve early recognition of the diseases in the preclinical stage but also allow for better differential diagnosis of the NDs. However, knowledge and awareness about factors influencing the aggregation, stability, and quantitative assessment of amyloid beta seems also to be pivotal in the aspect of variation in outcomes caused by this protein. Thus, the need for standardization in procedures of biological material preparation for Aβ assessment and applied methods is highlighted. Ongoing clinical trials have aimed at the development of new effective therapeutic options for NDs, particularly AD, which includes a broad spectrum of different agents, including immunotherapy against amyloid beta, tau protein, tau inhibitors, and anti-neuroinflammatory agents.

## Figures and Tables

**Table 1:** Hallmarks of AD pathology.

Pathological Hallmark	Comment	Ref
Tau proteins	In AD, tau undergoes abnormal hyperphosphorylation, leading to the formation of neurofibrillary tangles (NFTs) inside neurons. Tau pathology is closely associated with neuronal dysfunction and cell death in AD, and is a hallmark of the disease.	[8,9]
*APOE* gene	The *APOE* gene is a risk factor for AD. The *APOE* ε4 allele is the strongest genetic risk factor for late-onset AD, and individuals carrying this allele have an increased risk of developing the disease.	[6,8,9]
Indicators of synaptic dysfunction	Synaptic dysfunction is an early event in the pathogenesis of AD. Various synaptic proteins, including synaptophysin and PSD-95, neurogranin, are altered in AD brains and may serve as biomarkers of synaptic integrity and dysfunction. Measurements of those proteins in cerebrospinal fluid (CSF) or blood may provide insights into synaptic pathology in AD.	[10,11]
Inflammatory molecules	Proteins involved in the inflammatory response, such as cytokines (e.g., interleukin-1β, tumor necrosis factor-α), chemokines and complement proteins, are upregulated in AD brains and contribute to disease progression.	[6,12,13]
Neurotrophic factors	Neurotrophic factors, such as brain-derived neurotrophic factor (BDNF) and nerve growth factor (NGF), play crucial roles in neuronal survival and function. Dysregulation of neurotrophic signaling pathways has been implicated in AD pathogenesis, and alterations in these factors may serve as markers of neurodegeneration in the disease.	[14,15]

**Table 2:** Significance of Aβ peptide in different conditions.

Condition	Main Pathological Features	Clinical Utility of Aβ Peptide	Ref.
CAA	It is characterized by the accumulation of amyloid beta plaques in the walls of small blood vessels in the brain. It can lead to cognitive impairment, strokes, and other neurological symptoms.	Diagnosis of amyloid beta deposition in the brain is crucial for confirming CAA and guiding treatment decisions.	[27]
DLB	Typical neuropathological changes in the brain are Lewy bodies and abnormal protein deposits. Amyloid pathology often coexists with alpha-synuclein pathology in DLB patients.	Detecting amyloid beta deposition can aid in distinguishing DLB from other types of dementia and form appropriate management strategies.	[28]
FTD	Characterized by progressive degeneration of the frontal and temporal lobes of the brain. While FTD is primarily associated with tau protein pathology, amyloid beta deposition can also occur in some subtypes, such as FTD with motor neuron disease.	Identifying amyloid beta pathology can help in accurately diagnosing and subclassifying FTD.	[29]
DS	Individuals with Down Syndrome have a higher risk of developing Alzheimer’s disease due to the triplication of chromosome 21, which contains the gene encoding amyloid precursor protein (APP).	Amyloid beta pathology often manifests early in individuals with DS, making early diagnosis and intervention critical for managing cognitive decline and other associated symptoms.	[30]
TBI	Accumulation of amyloid beta plaques has been observed in the brains of individuals with a history of traumatic brain injury. Chronic Traumatic Encephalopathy (CTE), a neurodegenerative condition associated with repeated head trauma, is characterized by the deposition of amyloid beta and other abnormal proteins.	Detecting amyloid beta pathology can aid in understanding the long-term consequences of TBI and developing targeted interventions.	[31]

**Table 3:** Techniques of amyloid beta detection.

Detection Method	Principle	Detected Aβ	Sensitivity & Specificity	Clinical Application	Advantages	Limitations	Ref.
SIMOA (Single Molecule Array)	Single-molecule detection	Aβ40, Aβ42	Ultra-high sensitivity	Yes	Detects low Aβ levels in plasma	High cost of equipment and reagents	[36,40]
IP-MS (Immunoprecipitation-Mass Spectrometry)	Immunoprecipitation of Aβ followed by mass analysis	Aβ40, Aβ42, fragments	High specificity and sensitivity	Yes (clinical studies)	Can analyze different isoforms	Expensive, requires specialized equipment	[42]
SPR (Surface Plasmon Resonance)	Detection of refractive index changes upon Aβ binding	Aβ40, Aβ42	High sensitivity	No (mainly research)	No need for labeling	Requires expensive equipment	[43,44]
Nanoparticle-based sensors	Detection of conductivity or optical changes in nanoparticles	Aβ40, Aβ42	High sensitivity	No (preclinical research)	Fast and potentially low-cost	Still in development	[45]
PET (Positron Emission Tomography)	Radiolabeled ligand binding to amyloid plaques	Aβ deposits in the brain	High specificity, does not detect free Aβ	Yes	Non-invasive brain amyloid imaging	Expensive, radiation exposure	[35,48]
Nanowire FET (Field-Effect Transistor Sensors)	Conductivity change in nanowires upon Aβ binding	Aβ40, Aβ42	Ultra-high sensitivity, real-time detection	No (preclinical research)	Ultra-sensitive, label-free detection	Variability in fabrication	[46]
Nanomechanical Resonators	Frequency shift upon Aβ interaction	Aβ40, Aβ42	High sensitivity	No (experimental phase)	Detects extremely low concentrations	Limited customization of data analysis	[47]

**Table 5:** Therapies targeting tau pathology.

Agent	Administration	Mechanism of action	Indication	Clinical Status	Ref.
Lithium	Oral	Neurotransmitter receptors ion channel modulator—improves neuropsychiatric symptoms	Mild to moderate AD	Phase II	[60,74]
Methylene Blue	Oral	MB inhibits the aggregation and misfolding of tau proteins	Mild to moderate AD	Phase II	[85]
Curcumin	Oral	Has shown potential as a tau aggregation inhibitor	Mild to moderate AD	Phase II	[86]
AADvac1	Subcutaneous (SC) injection	Anti-tau active vaccine, stimulates the immune system to produce antibodies against phosphorylated tau (pTau)	Early-stage Alzheimer’s disease or mild cognitive impairment (MCI)	Phase II	[87]
ACI-35	Subcutaneous (SC) injection	Anti-tau active vaccine, stimulates the immune system to produce antibodies against phosphorylated tau (pTau)	Early-stage Alzheimer’s disease or mild cognitive impairment (MCI)	Phase Ib/IIa	[88]
Gosuranemab	Intravenous (IV) infusion	Monoclonal antibody (mAb) targeting tau protein	Early-stage Alzheimer’s disease or mild cognitive impairment (MCI)	Phase II	[89]

**Table 6:** Other methods of treatment tested for AD targeting neuroinflammation, insulin resistance and microbiome pathology.

Agent	Administration	Mechanism of Action	Indication	Clinical Status	Ref.
CSF1R inhibitors	Oral	Blocking microglial proliferation	AD, Frontotemporal Dementia, Parkinson’s Disease	Phase I/II	[93,94]
Stattic	Intraperitoneal (IP) injection or oral gavage	Selective STAT3 inhibitor	Mild cognitive impairment and AD	Preclinical	[95]
Insulin therapy	Intranasal	Improving brain glucose metabolism, reducing neuroinflammation, and potentially modulating amyloid beta and tau pathologies	Mild cognitive impairment and AD	Phase II, III	[96]
GLP-1	Subcutaneous injection, oral (Rybelsus)	Neuroprotective effects by promoting neuronal survival, enhancing synaptic plasticity, and supporting neurogenesis, reducing neuroinflammation and oxidative stress, reducing tau hyperphosphorylation and amyloid beta accumulation	Mild to moderate AD	Phase II: Liraglutide and Exenatide, phase III: Semaglutide	[97]
GIP	Subcutaneous injection	Activation of GIP receptors in the brain has been shown to exert neuroprotective effects, enhancing neurogenesis, synaptic plasticity, and cognitive function. Indirect effects on Aβ accumulation, a hallmark of Alzheimer’s disease	Mild-to-moderate AD, Parkinson’s Disease	Preclinical, moving into phase I and phase II trials	[98]
Metformin	Oral	Improving brain glucose metabolism, metformin may help preserve cognitive function and slow the progression of AD. Also activates AMPK, a cellular enzyme that regulates energy balance and reduces neuroinflammation	Mild-to-moderate AD	Phase II trials for AD, with potential phase III trials depending on results	[99,100]
Pioglitazone	Oral tablets	PPAR-γ activation reduces neuroinflammation, improves insulin sensitivity, enhances brain glucose metabolism, reduces amyloid and tau aggregation	Alzheimer’s disease (mild to moderate), mild cognitive impairment, type 2 diabetes with cognitive decline	Preclinical, phase II trials in AD, with potential phase III studies depending on results	[101,102]
Sodium oligomannate	Oral tablets	Modulates gut microbiota, reduces neuroinflammation, enhances amyloid beta clearance, improves immune function	Alzheimer’s disease (mild to moderate), potential for other neurodegenerative diseases	Phase II (completed), phase III (completed in China), approved in China for AD	[103]
